# Supplementary material for: New Chemicals Suppressing SARS-CoV-2 Replication in Cell Culture
Source: Molecules. 2022 Sep 5;27(17):5732. doi: 10.3390/molecules27175732 (PMC9457583; doi:10.3390/molecules27175732)
Supplement: Supplementary file 1 [file molecules-27-05732-s001.zip › molecules-1894619-supplementary.pdf]

molecules

New Chemicals Suppressing SARS-CoV-2 Replication in Cell Culture

Alexey Sulimov [1,2], Ivan Ilin [1,2], Danil Kutov [1,2,*] , Khidmet Shikhaliev [3], Dmitriy Shcherbakov [4], Oleg Pyankov [4], Nadezhda Stolpovskaya [3], Svetlana Medvedeva [3] and Vladimir Sulimov [1,2,*]

1 Dimonta Ltd., 15 Nagornaya Str., Bldg 8, 117186 Moscow, Russia
2 Research Computing Center, Lomonosov Moscow State University, Leninskie Gory, 1, Building 4, 119234 Moscow, Russia
3 Department of Organic Chemistry, Faculty of Chemistry, Voronezh State University, 1 Universitetskaya Sq., 394018 Voronezh, Russia
4 State Research Centre of Virology and Biotechnology "Vector", 630559 Koltsovo, Russia
* Correspondence: dk@dimonta.com (D.K.); vladimir.sulimov@gmail.com (V.S.)

Contents:

1. NMR Spectra S2

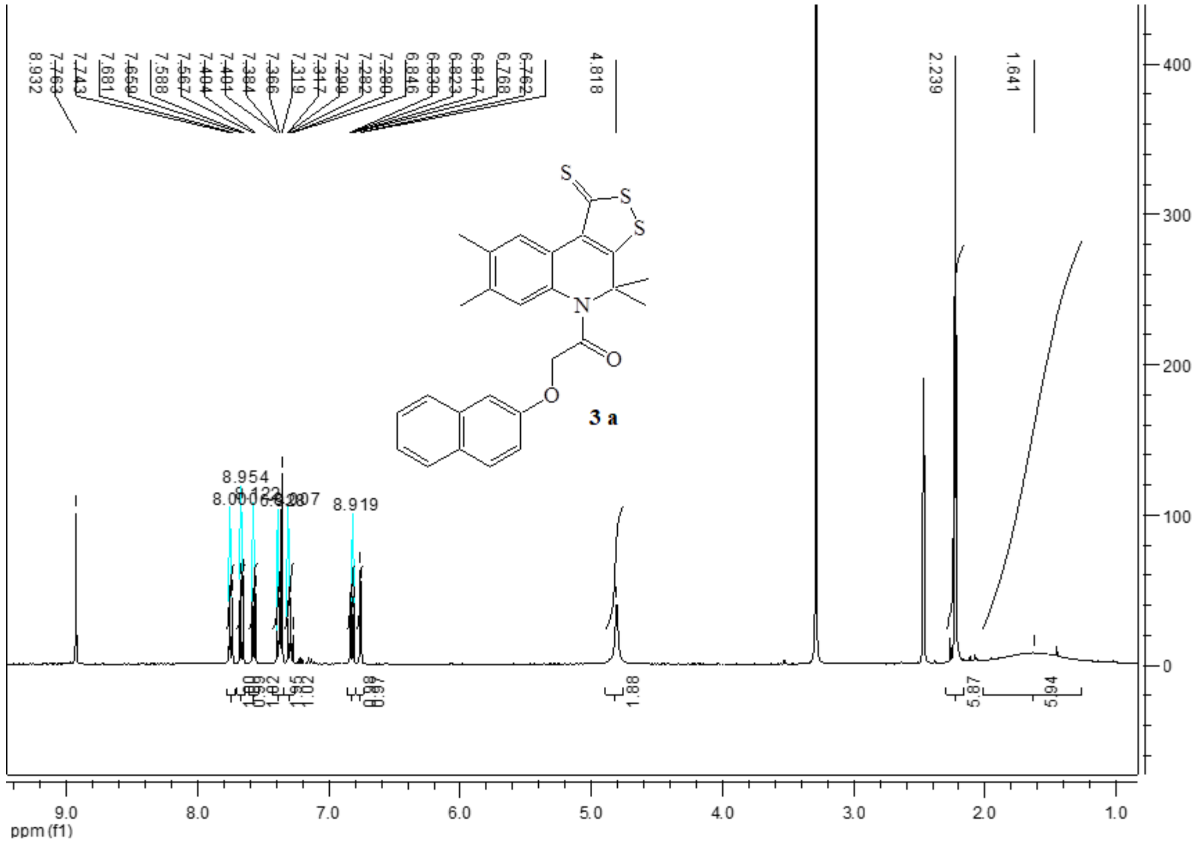

Figure S1. ^{1}H NMR spectra of compound **3a**.

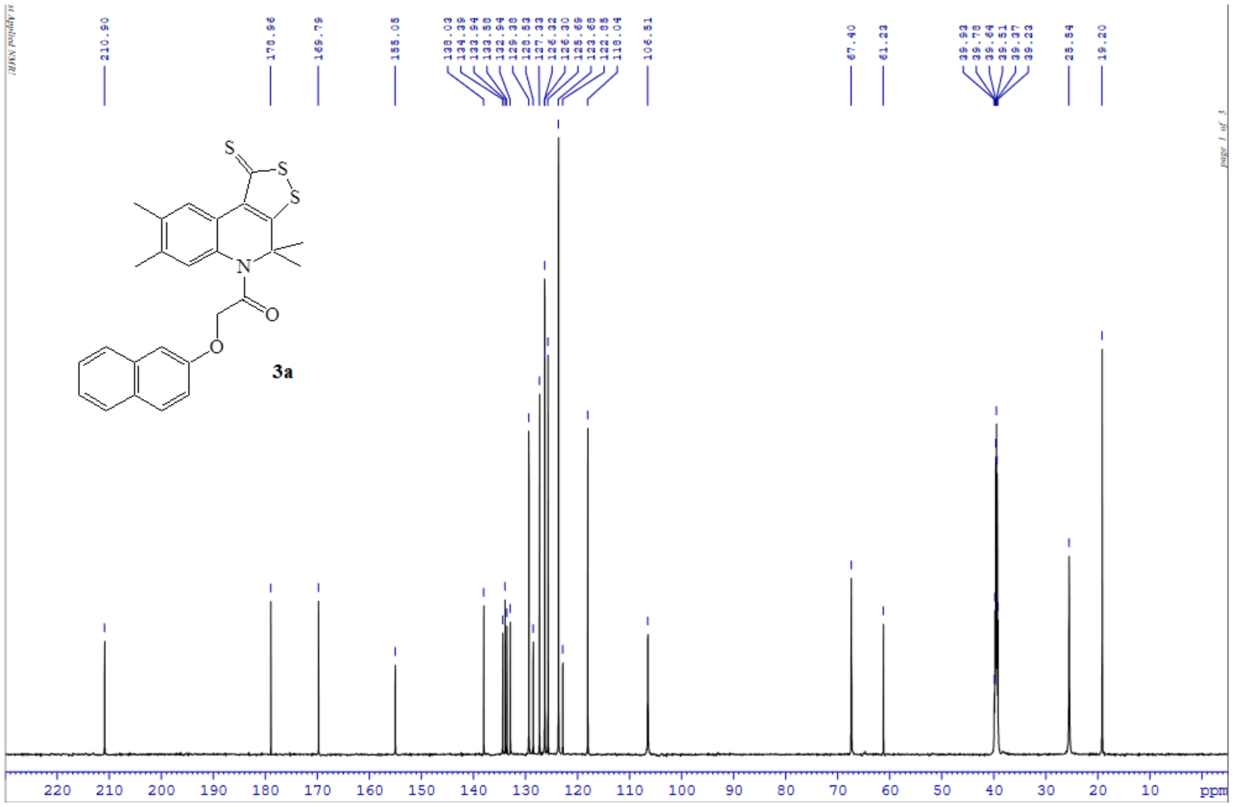

Figure S2. ^{13}C NMR spectra of compound **3a**.

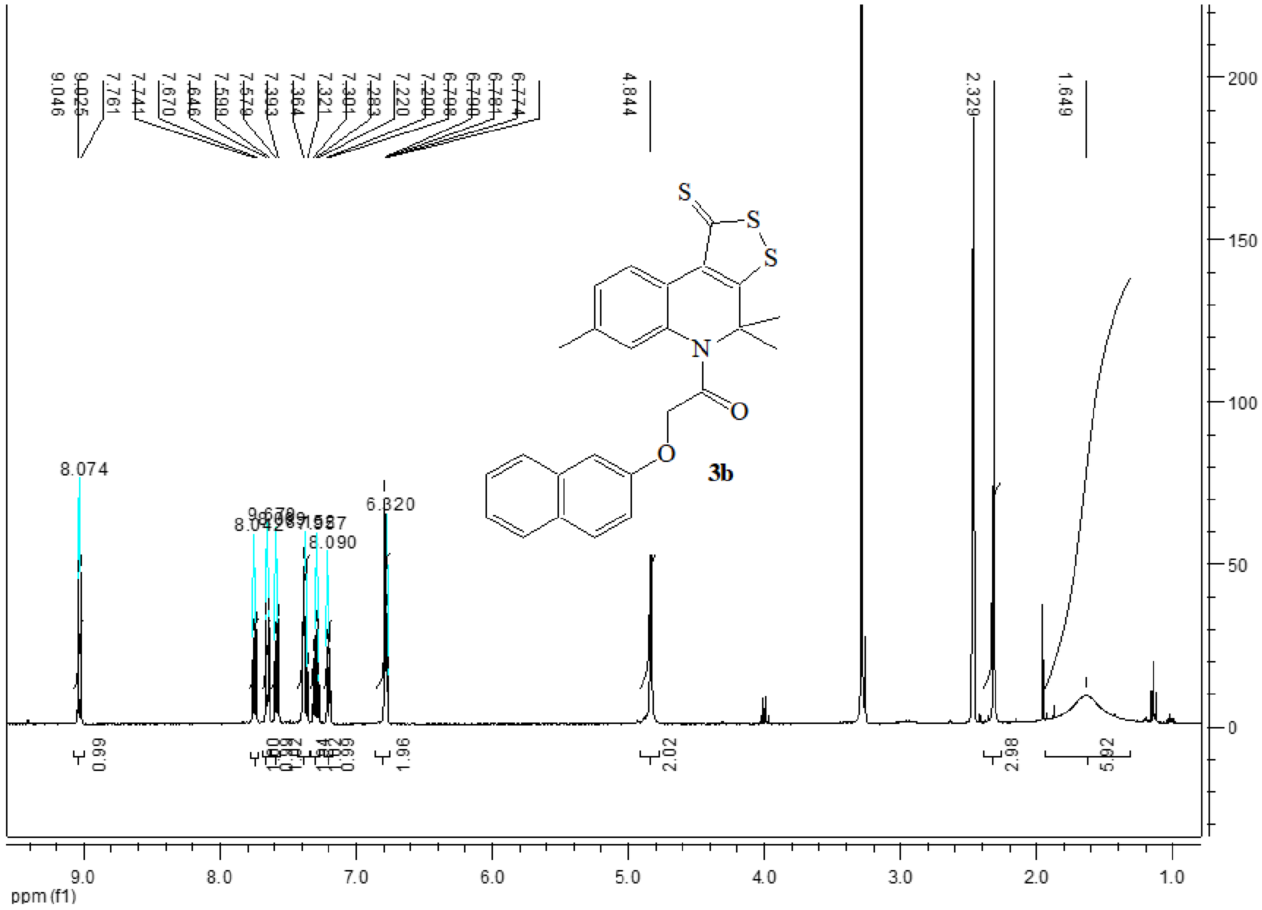

Figure S3. ^{1}H NMR spectra of compound **3b**

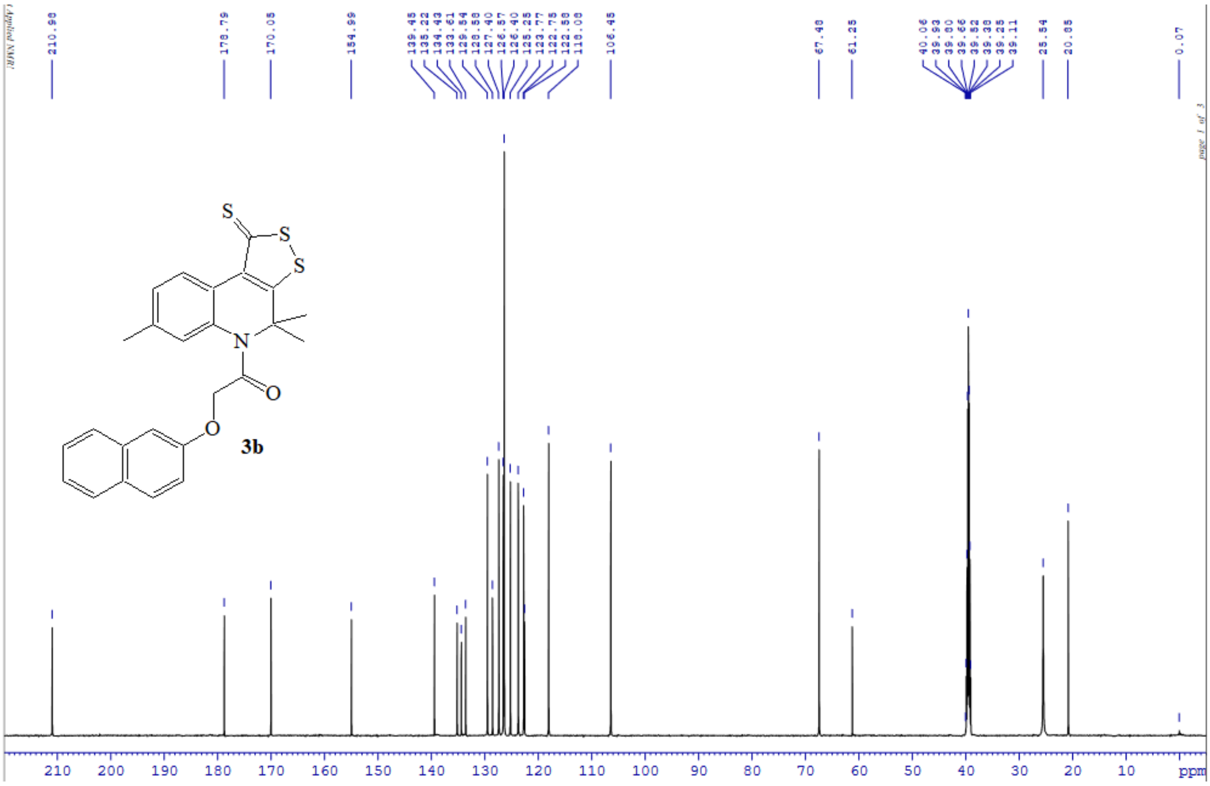

Figure S4. ^{13}C NMR spectra of compound **3b**

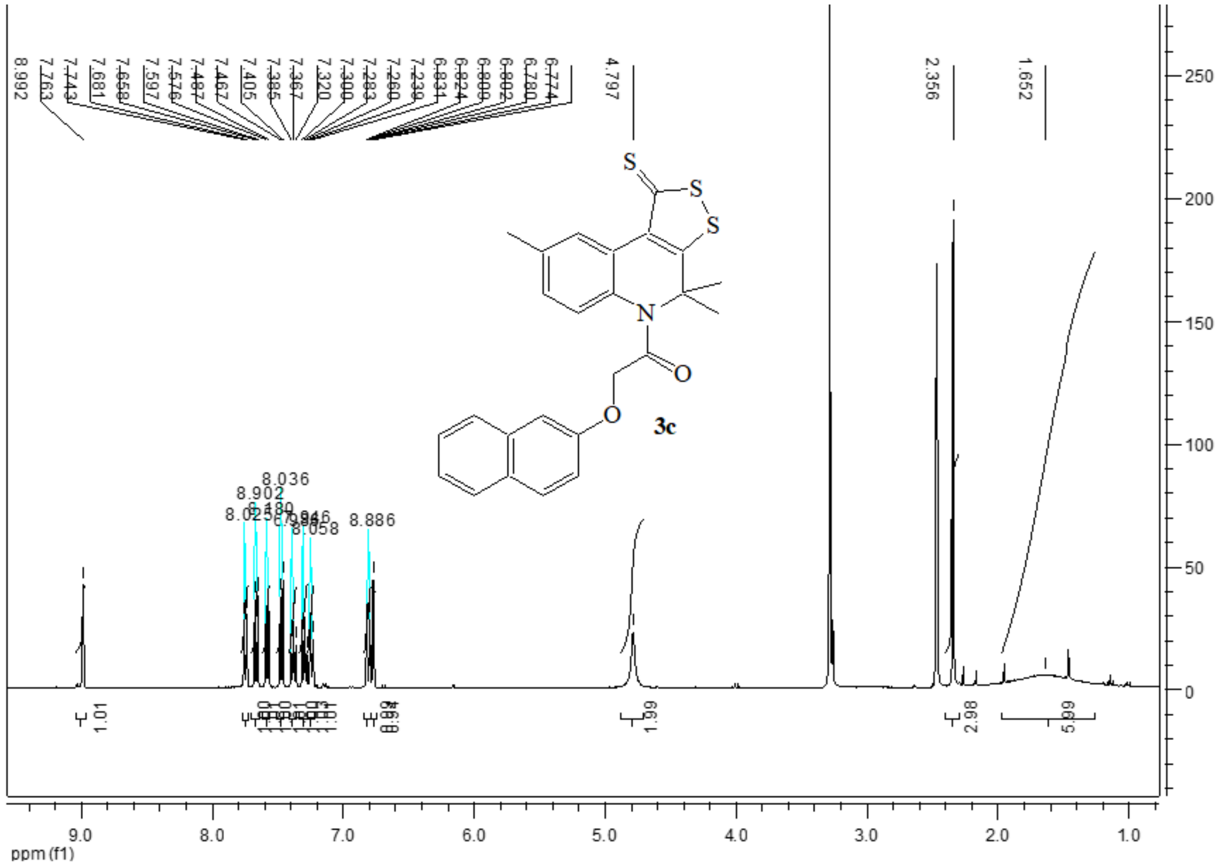

Figure S5. ^{1}H NMR spectra of compound **3c**

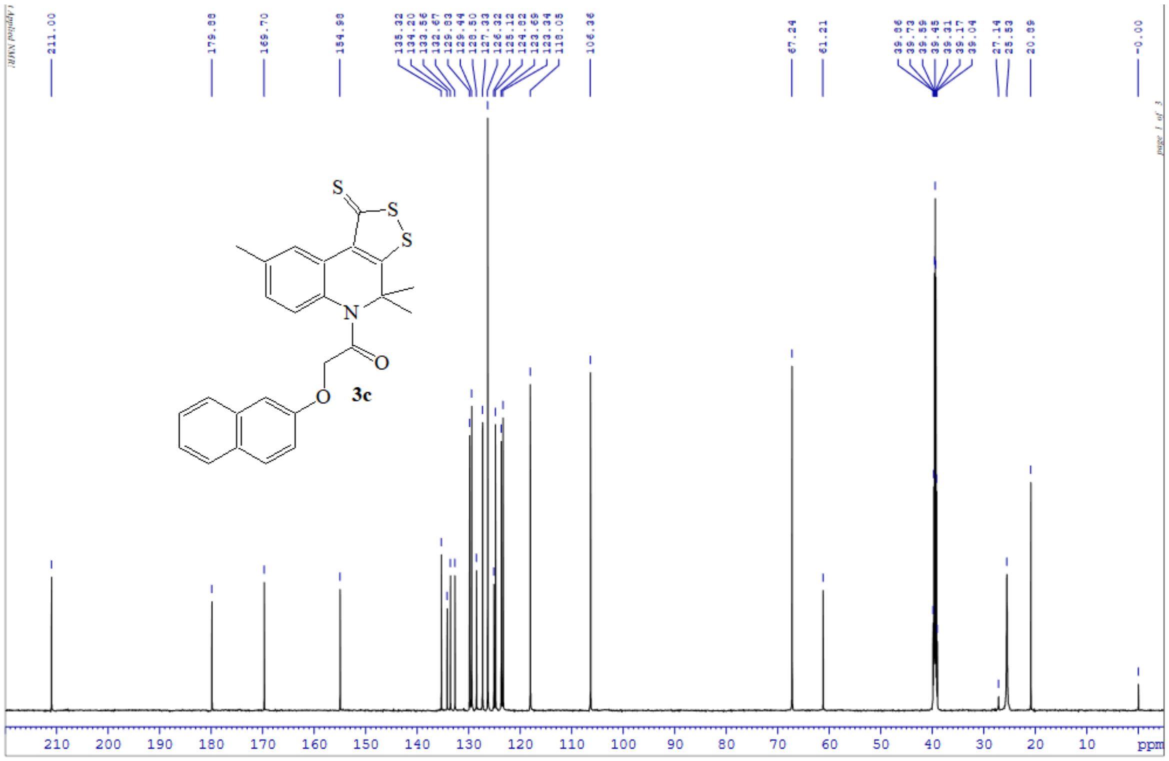

Figure S6. ^{13}C NMR spectra of compound **3c**

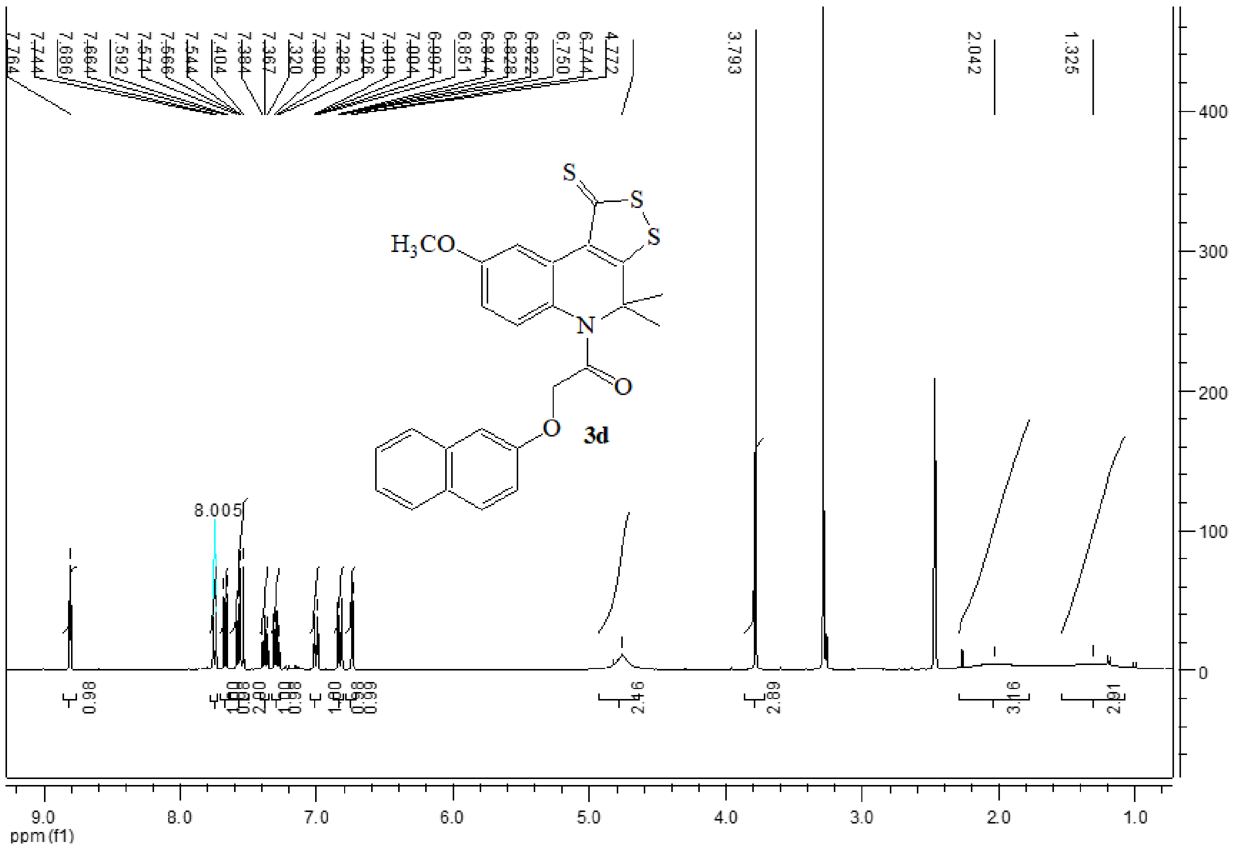

Figure S7. ^{1}H NMR spectra of compound **3d**

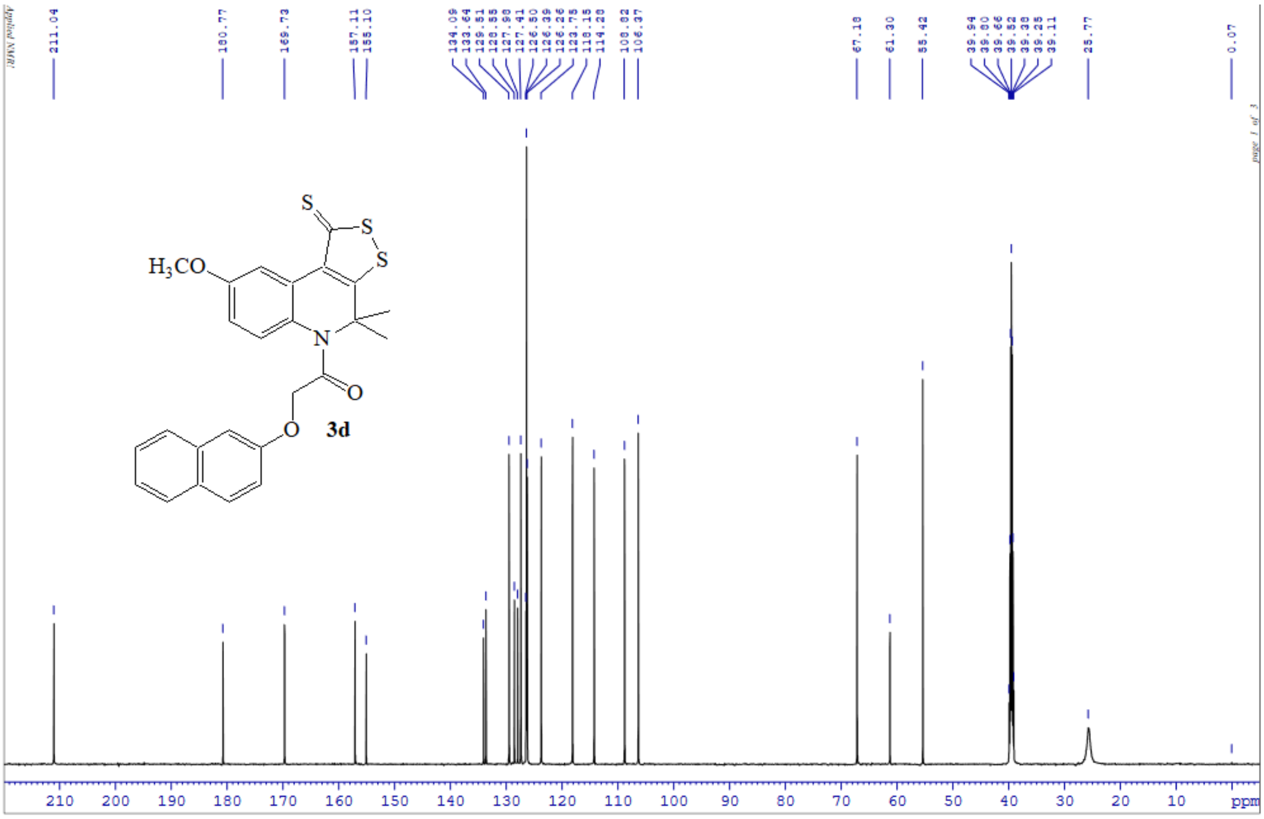

Figure S8. ^{13}C NMR spectra of compound **3d**

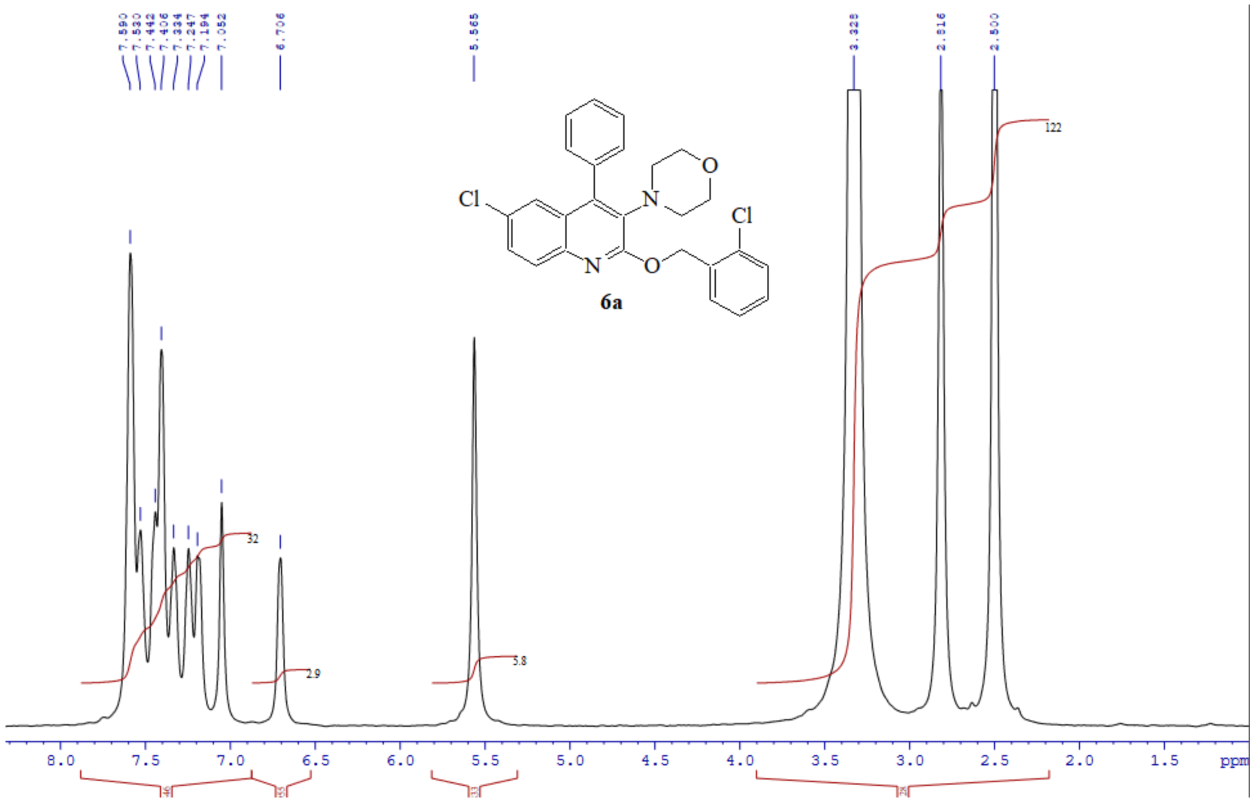

Figure S9. ^{1}H NMR spectra of compound **6a**

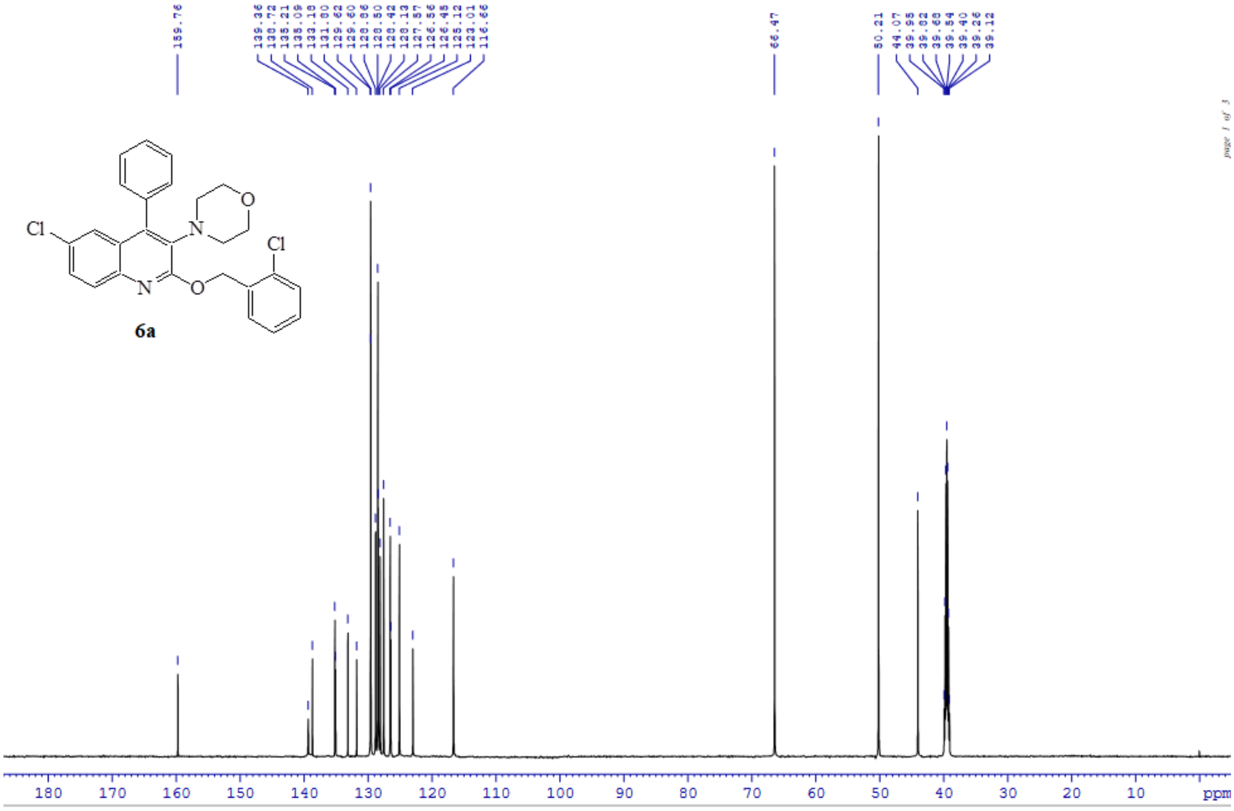

Figure S10. ^{13}C NMR spectra of compound **6a**

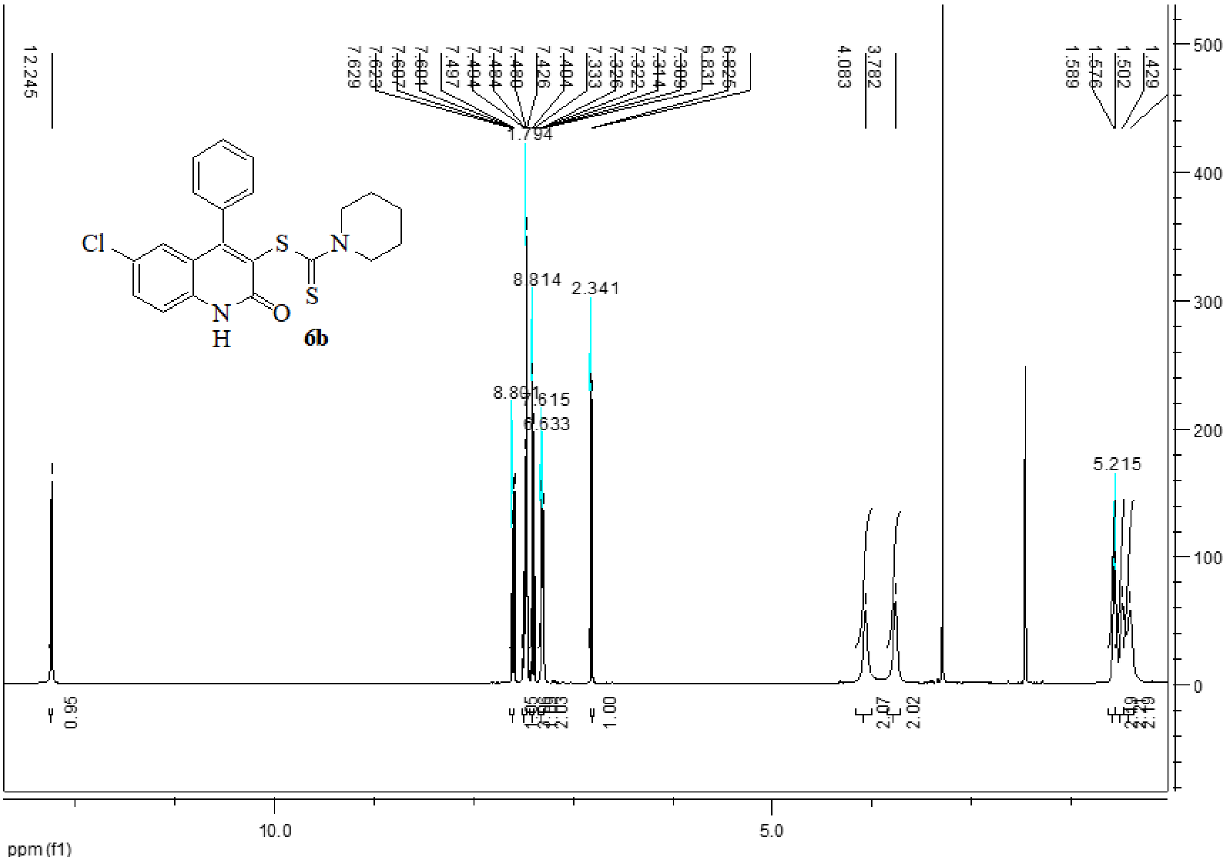

Figure S11. ^{1}H NMR spectra of compound **6b**

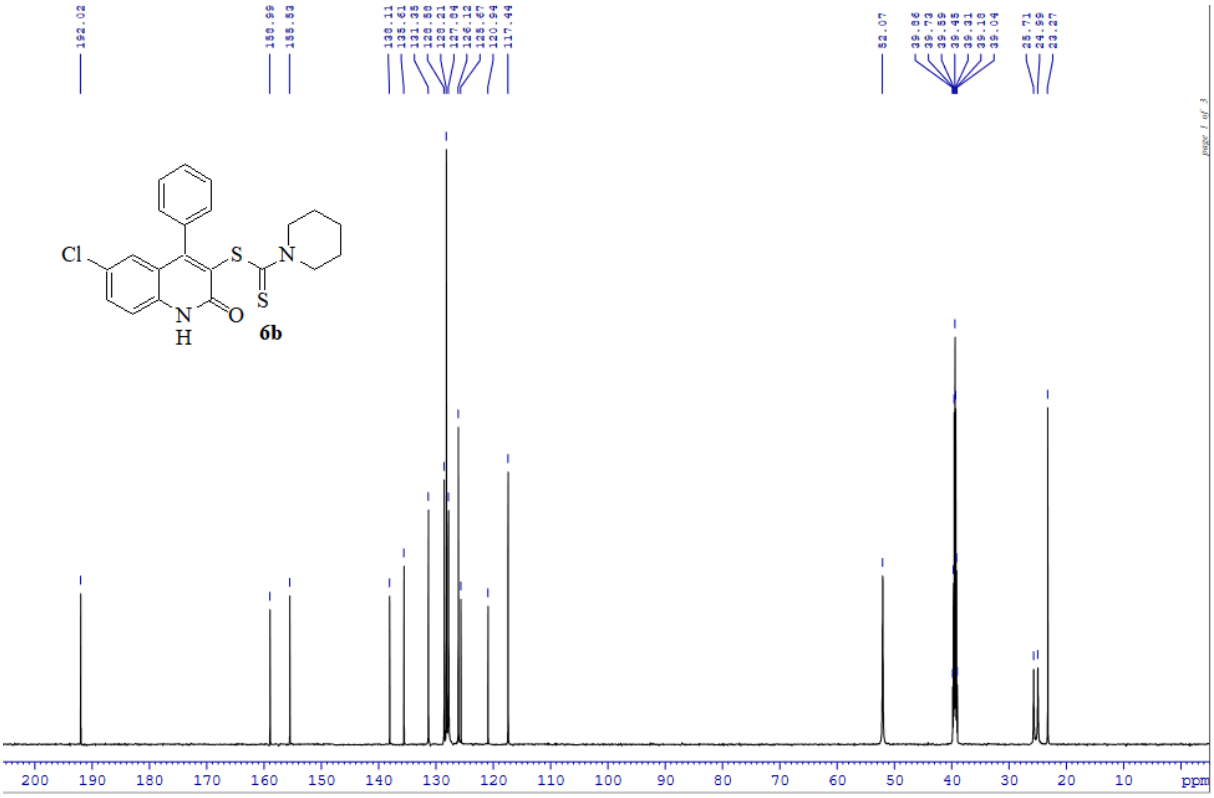

Figure S12. ^{13}C NMR spectra of compound **6b**

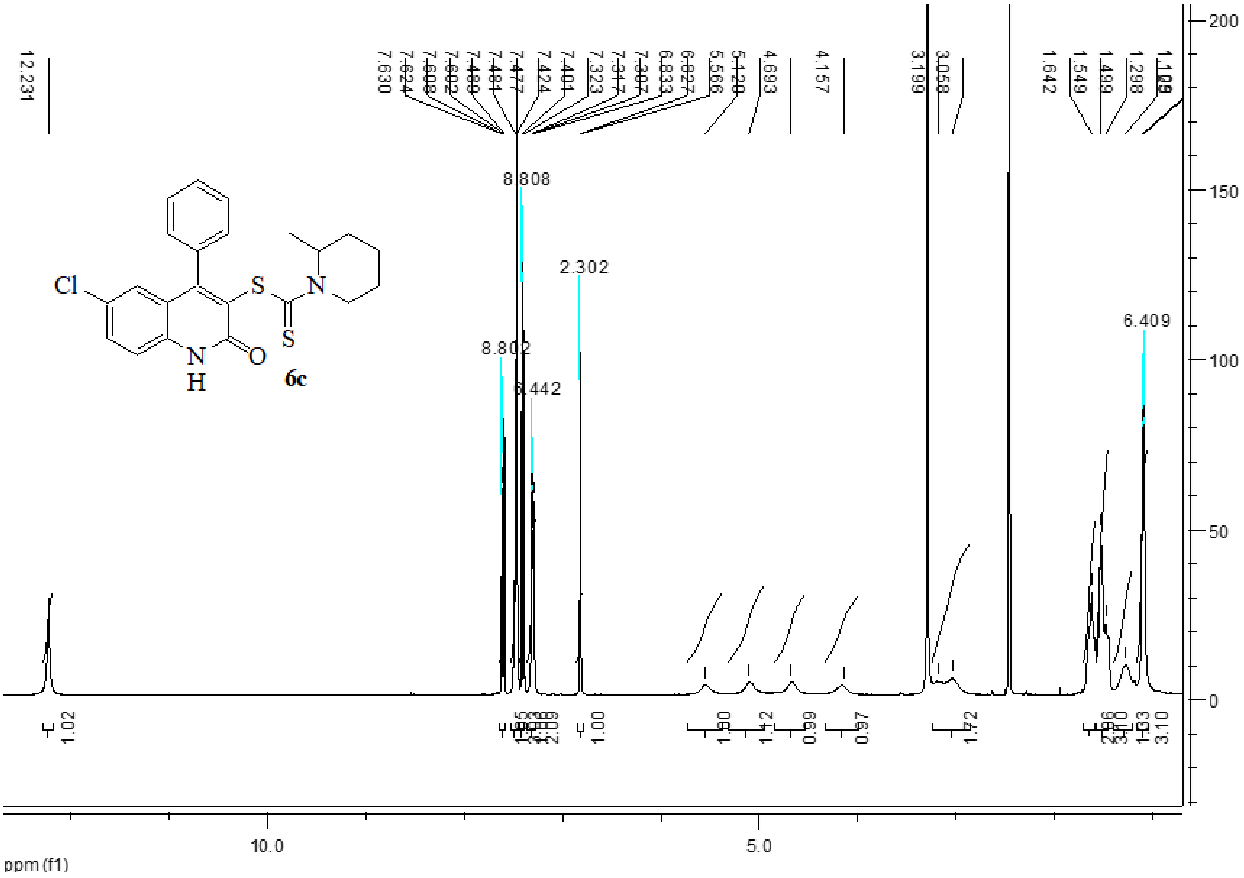

Figure S13. ^{1}H NMR spectra of compound **6c**

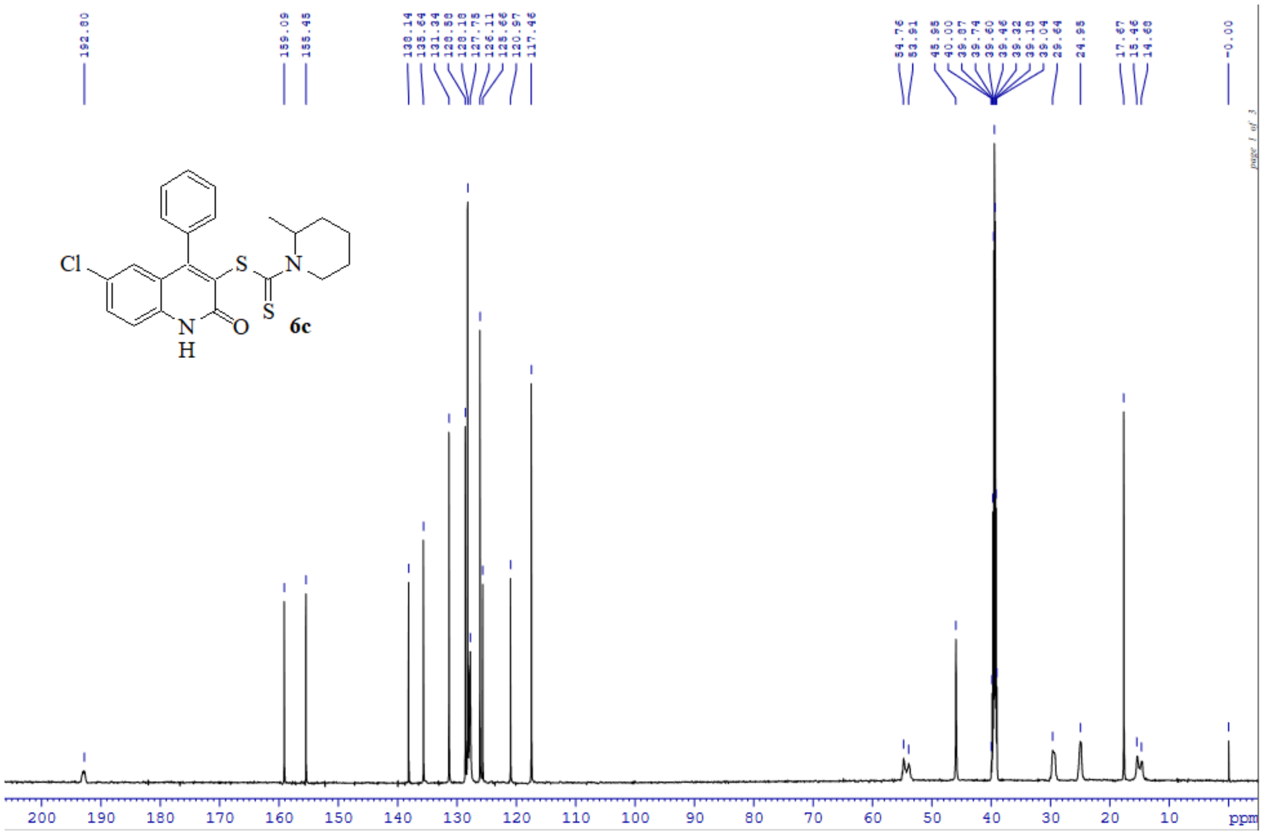

Figure S14. ^{13}C NMR spectra of compound **6c**

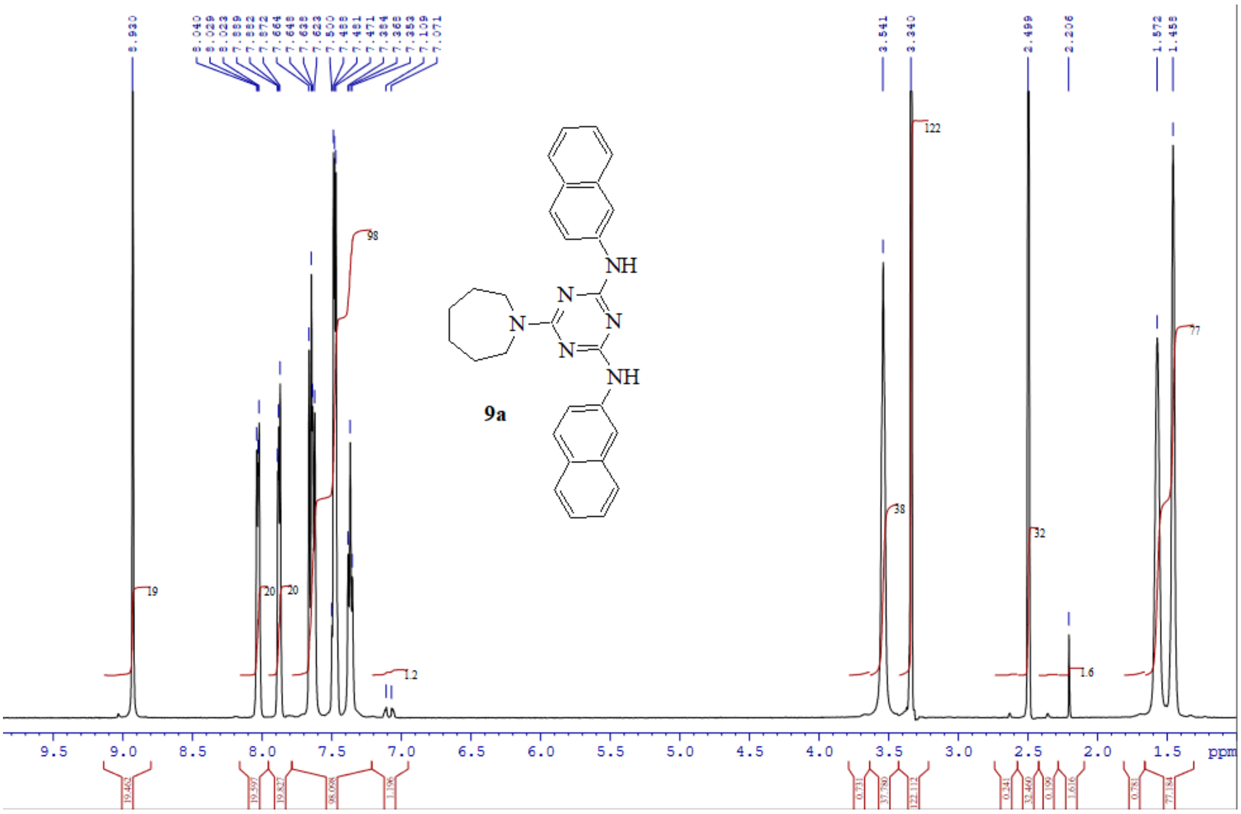

Figure S15. ^{1}H NMR spectra of compound **9a**

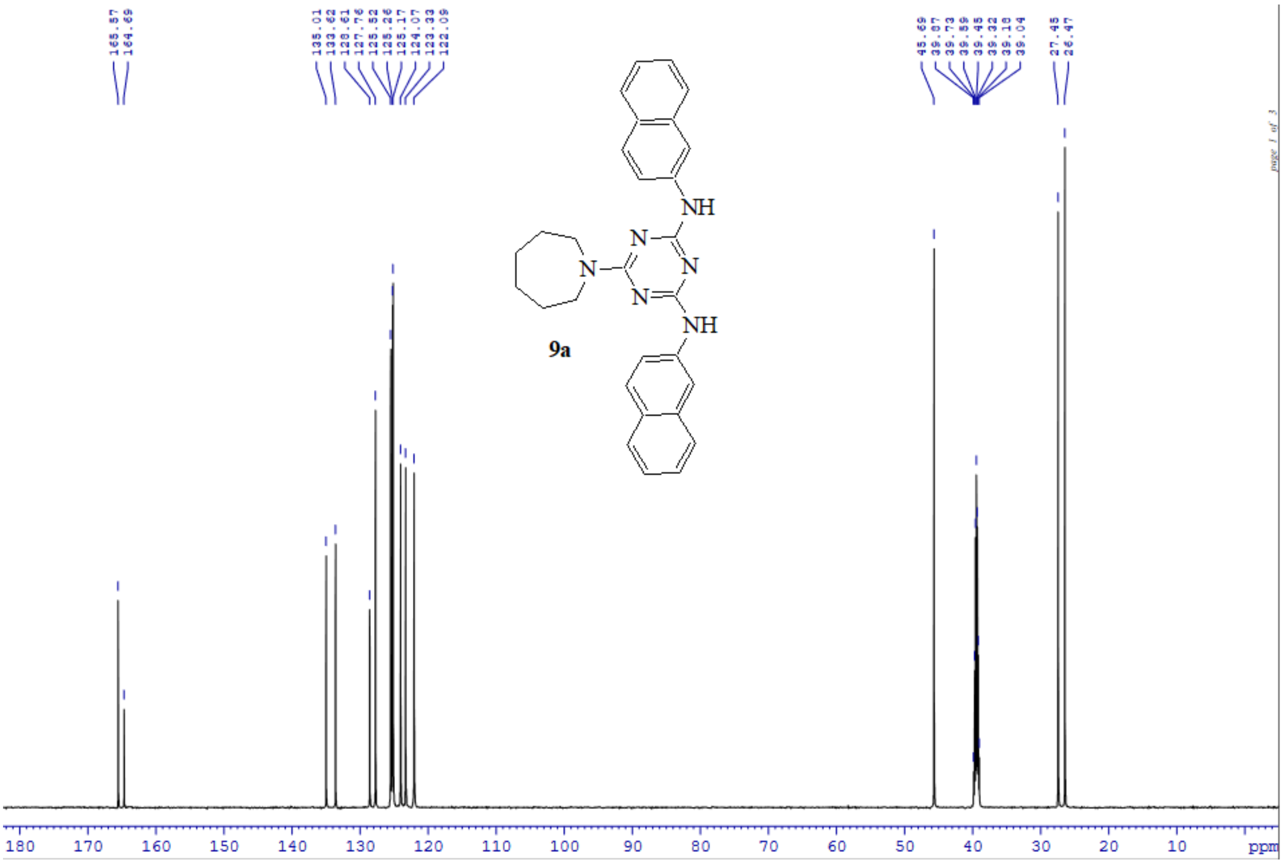

Figure S16. ^{13}C NMR spectra of compound **9a**

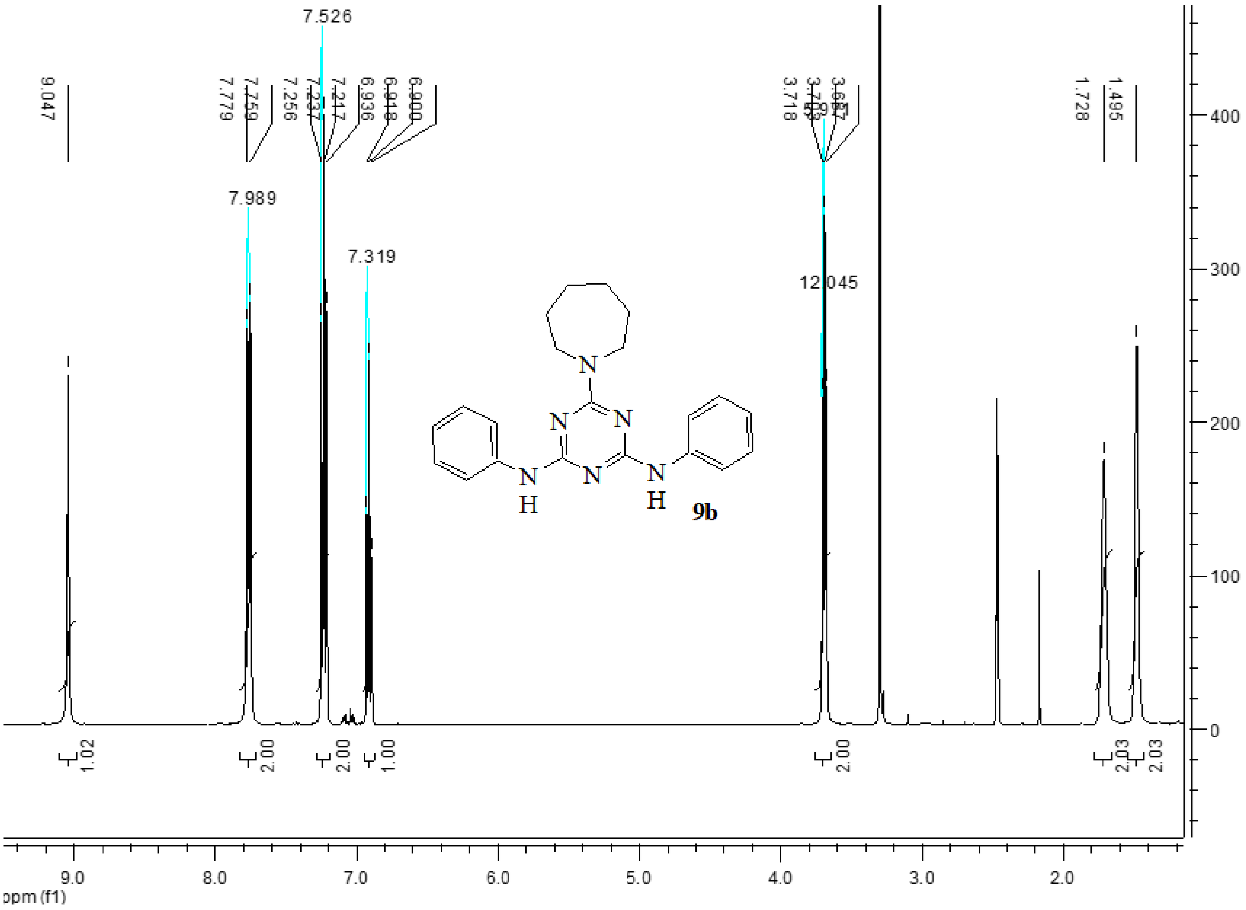

Figure S17. ^{1}H NMR spectra of compound **9b**

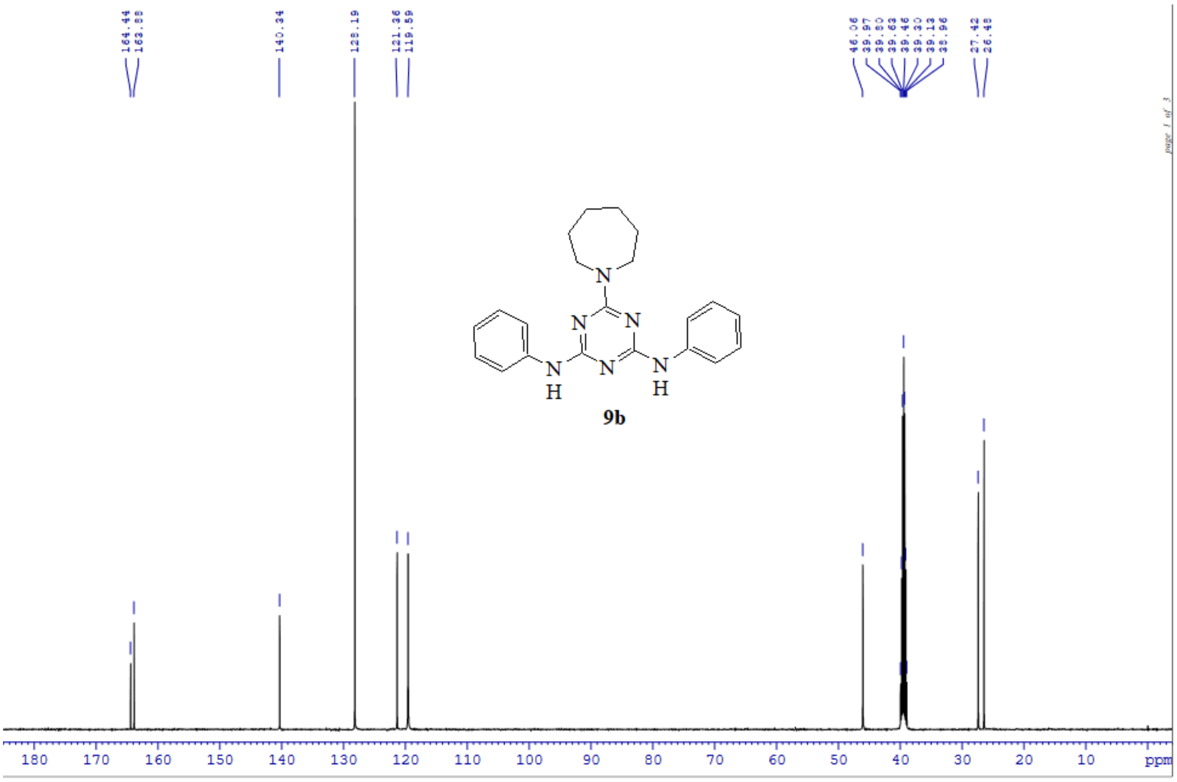

Figure S18. ^{13}C NMR spectra of compound **9b**

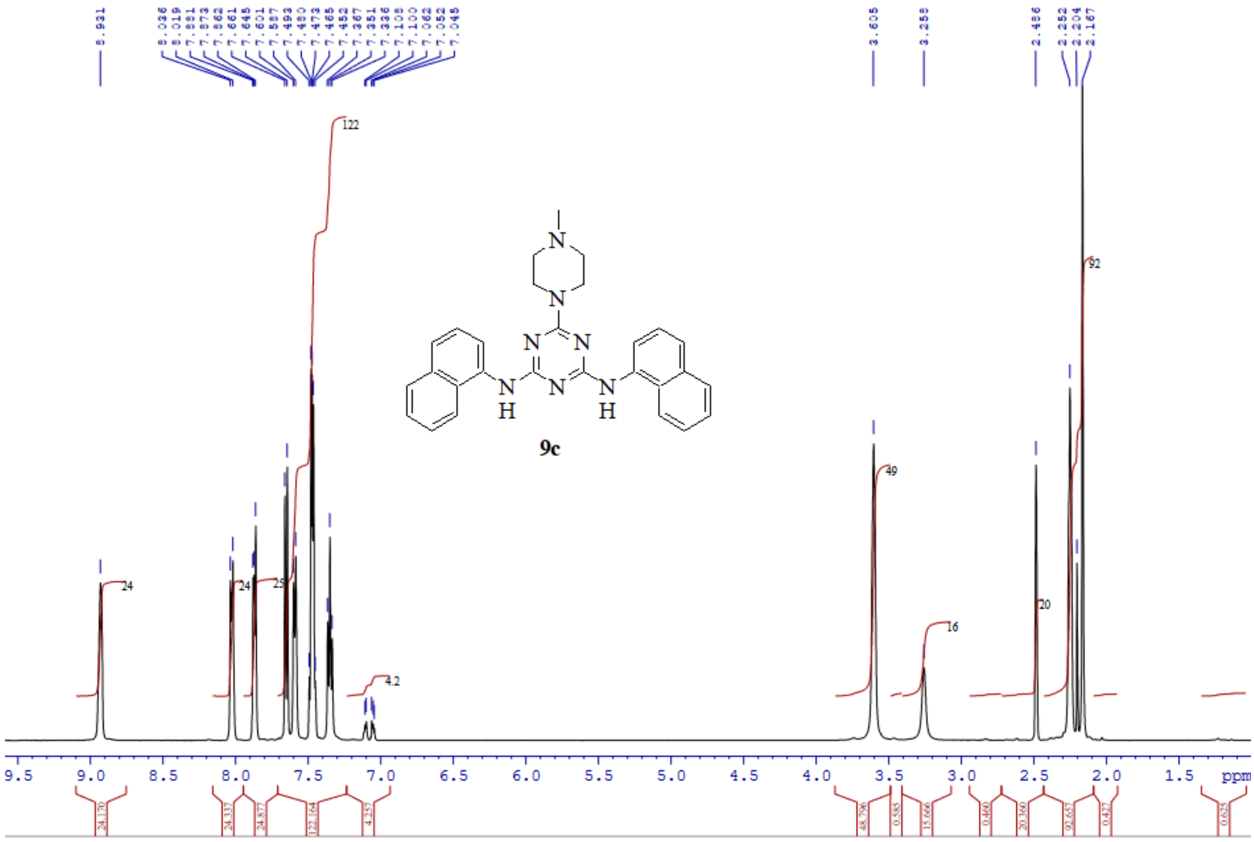

Figure S19. ^{1}H NMR spectra of compound **9c**

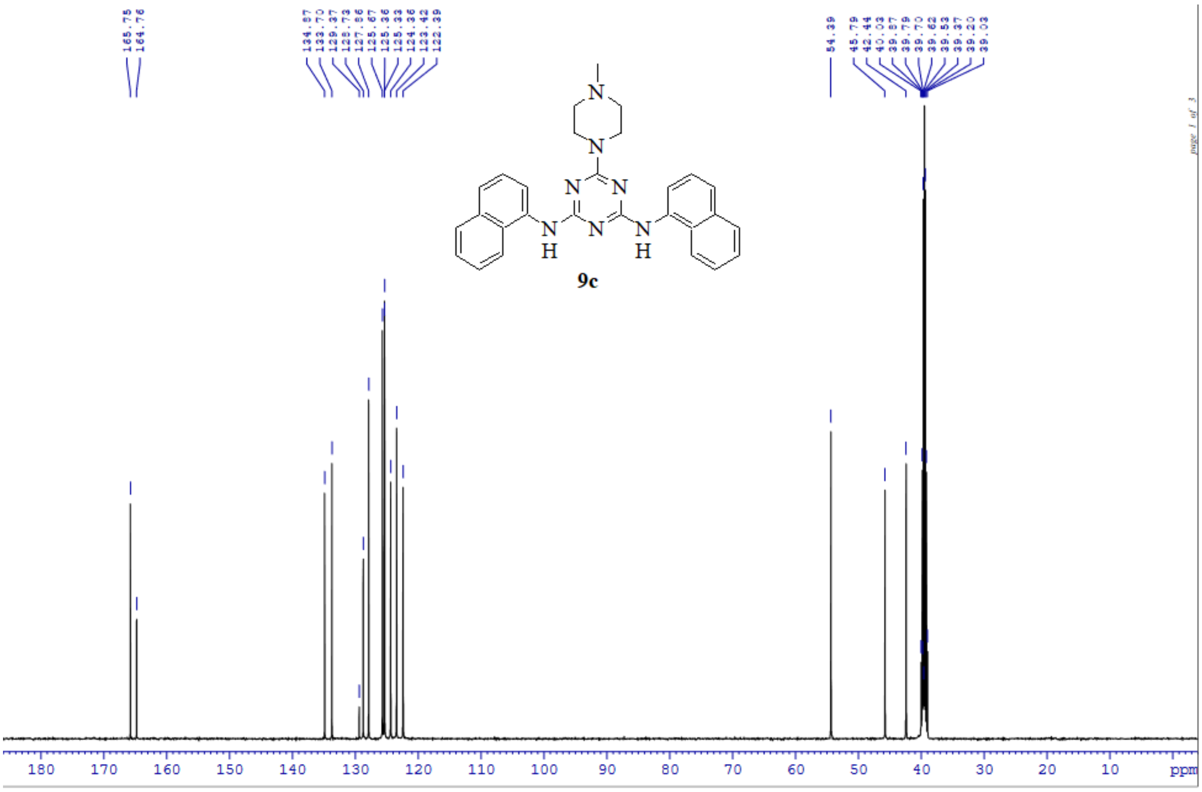

Figure S20. ^{13}C NMR spectra of compound **9c**
